# The murine cerebral malaria phenomenon

**DOI:** 10.1016/j.pt.2009.10.007

**Published:** 2010-01

**Authors:** Nicholas J. White, Gareth D.H. Turner, Isabelle M. Medana, Arjen M. Dondorp, Nicholas P.J. Day

**Affiliations:** 1 Mahidol Oxford Research Unit, Faculty of Tropical Medicine, Mahidol University, Bangkok, Thailand; 2 Centre for Tropical Medicine, Churchill Hospital, Oxford, UK; 3 Malaria Research Group, Nuffield Department of Clinical and Laboratory Sciences, John Radcliffe, Hospital, Oxford, UK

## Abstract

*P.berghei* ANKA infection in CBA or CB57BL/6 mice is used widely as a murine ‘model’ of human cerebral malaria (HCM), despite markedly different histopathological features. The pathology of the murine model is characterised by marked inflammation with little or no intracerebral sequestration of parasitised erythrocytes, whereas HCM is associated with intense intracerebral sequestration, often with little inflammatory response. There are now more than ten times as many studies each year of the murine model than on HCM. Of 48 adjunctive interventions evaluated in the murine model, 44 (92%) were successful, compared with only 1 (6%) of 17 evaluated in HCM during the same period. The value of the mouse model in identifying pathological processes or therapeutic interventions in human cerebral malaria is questionable.

## Cerebral malaria – in mice and men

Obtundation and stupor are usual in the agonal phase of any acute lethal infection, and can occur transiently in many febrile infections including malaria, but the syndrome of human cerebral malaria (HCM) is distinct. Most (80–85%) patients survive, and most have no evident residual ‘structural’ neurological deficits [1]. HCM is a major cause of death in tropical countries, and yet there have been remarkably few pathological studies of severe falciparum malaria, particularly from Africa, where the majority of deaths occur [2]. The only interventions that have reduced the mortality of severe falciparum malaria are antimalarial drugs and haemofiltration for malaria-associated acute renal failure [3,4]. Many adjunctive treatments have been tried that were based on the prevailing pathophysiological hypotheses of the time. None was clearly beneficial, although most clinical studies were insufficiently powered to detect anything but dramatic reductions in mortality. Several interventions (heparin, dexamethasone, phenobarbitone, anti-TNF antibody) appeared harmful [5–7]. In recent years, clinical trial networks have been established, and large clinical trials have been conducted in adults and children with severe falciparum malaria, powered to show reductions in mortality of one-third or less [3,8–10]. Large trials take time and are costly. Probably only one or two can be conducted in the world at any time, so the choice of the intervention and the design of the trial are crucial. These networks could soon be used to evaluate adjunctive treatments or other aspects of supportive care – but which ones?

**Figure 1:**
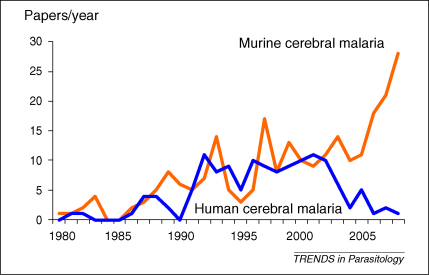
Annual numbers of publications describing studies on human cerebral malaria (HCM) (blue) and murine models of cerebral malaria (orange) since 1980 listed on the National Library of Medicine (NLM) PubMed. The medical subject headings (MeSH) search terms were: human/cerebral malaria; *subheadings*, therapy, drug therapy, diet therapy/physiopathology, pathology; *limits*, concerning therapy: clinical trials. MeSH: mouse/cerebral malaria; *subheadings*, therapy, drug therapy, diet therapy/physiopathology, pathology; *limits*, none. After compilation, the lists were checked manually.

Over the past 20 years there has been a remarkable increase in the number of laboratory-based studies in temperate countries on a murine ‘model’ of cerebral malaria involving *Plasmodium berghei* (ANKA strain) infection of C57BL/6 or CBA mice. Instead of dying slowly from anaemia, mice with this particular infection die more rapidly (in the second week of infection) from a cerebral syndrome associated with seizures. This is a specific model, requiring a specific combination of parasite and host, because infections with the majority of *P. berghei* strains, and in most of the other strains of mice tested, do not cause a lethal encephalopathy. So what do the results of these numerous murine studies tell us about the infection in humans? (There are now over ten times more murine studies per year than there are studies on HCM) (Figure 1). And how can they be used to guide our therapeutic assessments? These are legitimate questions in that most grant applications to support research on murine cerebral malaria start with, and most papers on the ‘model’ conclude with, a statement that the results of the studies are of relevance to either the pathogenesis or the treatment of human cerebral malaria.

## The pathology of human cerebral malaria

**Figure 2:**
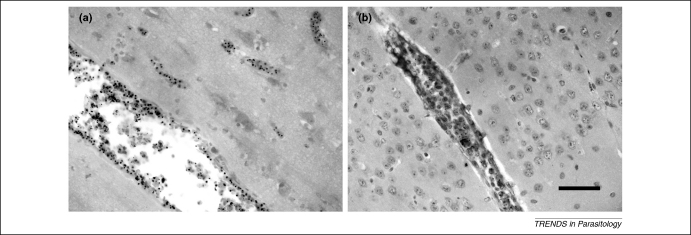
A comparison of the histopathological hallmarks of human cerebral malaria (HCM) and the *Plasmodium berghei* ANKA murine model. **(a)** Parasitised erythrocyte sequestration in small and large vessels from a patient who died during the acute phase of cerebral malaria. **(b)** A large vessel containing numerous mononuclear leukocytes but no sequestered parasitised erythrocytes from a mouse at the terminal stage of the disease. Sections are from cerebral cortex counterstained with haematoxylin. Scale bar, 50 μm.

The seminal pathology studies of Marchiafava and his colleagues at the end of the 19^th^ century [11] showed that red blood cells (RBCs) containing mature stages of *Plasmodium falciparum* malaria parasites are sequestered in the capillaries and post-capillary venules of the brain (Figure 2), and reduce the vascular lumen to create a mechanical obstruction to RBC transit. This was later shown to result from specific adherence of parasitised RBCs to the vascular endothelium (cytoadherence). Parasitised RBCs also adhere to uninfected RBCs (rosetting) and to other infected RBCs (auto-agglutination). In severe malaria (but not in sepsis) the entire RBC population becomes rigid, further compromising flow through capillaries that have a mid-point diameter smaller than the erythrocyte itself [12]. Although there are similarities between the clinical presentations of severe malaria and bacterial septicaemia, and the two commonly co-exist, the fundamental microvascular pathology is different [13]. Disease severity in severe falciparum malaria is proportional to the estimated sequestered parasite biomass [14]. The importance of tissue hypoxia is reflected in the reproducible observation that hyperlactataemia, with an increased lactate:pyruvate ratio, and acidosis are among the strongest prognostic indicators in adults and children with severe malaria [15–17]. The degree of microvascular obstruction and reduction in blood flow assessed directly *in vivo* correlates with the degree of hyperlactataemia [13]. Coma is associated quantitatively with cerebral sequestration [18]. In other words, patients who die from HCM have more parasitised erythrocyte sequestration in the cerebral microvasculature than do those who die from dysfunction of other vital organs. In some fatal cases, cerebral sequestration is not evident. These patients often had received antimalarial treatment for several days and cleared most intracerebral parasites (although residual adherent erythrocyte membranes and pigment commonly remain, testifying to earlier and more intense intracerebral sequestration). There are other causes of coma, such as status epilepticus [19] or a post-ictal state; indeed, the diagnosis of cerebral malaria could be incorrect, and coma could have a different causation [2]. Severe malaria is certainly overdiagnosed in endemic areas where the majority of the population is parasitised [20]. Our interpretation of the combined pathological evidence is that intracerebral sequestration of parasitised erythrocytes causes coma, and is therefore the primary cause of HCM, although the relative contributions of endothelial activation and dysfunction, reversible axonal dysfunction, and other local and systemic factors are not known [21–23]. There has been considerable debate as to the extent and importance of inflammatory changes in the brain in HCM [2,24–26]. Protagonists point to systemic pro-inflammatory cytokine responses (as in most febrile infections), evidence of platelet and some leukocyte accumulation in the cerebral microvasculature, particularly in children [27], and a moderate increase in blood–brain barrier permeability. Others point to the complete absence of inflammatory changes in many patients dying in the acute phase of cerebral malaria with a cerebral microvasculature engorged by cytoadherent parasitised erythrocytes [18,24,28–30].

## Animal ‘models’ of cerebral malaria

Ideally, prospective interventions should be tested in an animal model before being evaluated in the corresponding human disease. Unfortunately there is no good animal model that reproduces both the clinical and pathophysiological features of cerebral malaria in humans. *Aotus* and *Saimiri* monkeys and chimpanzees can be infected with *P. falciparum,* and both *P. coatneyi* and *P. fragile* infections in macaque monkeys are associated with sequestration of parasitised RBCs in the microvasculature, although cerebral sequestration is not prominent [31–33]. A rodent model of cerebral malaria was first proposed 35 years ago [34]. In recent years, infections with *P. berghei* ANKA strain in C57BL/6 or CBA mice have been studied almost exclusively. The murine malaria encephalopathy is very different from human cerebral malaria. It has an unequivocally immunological basis in which pro-inflammatory cytokines play a central role. *P. berghei* ANKA-infected murine erythrocytes do sequester to some extent in the microvasculature of mice, via adhesion to CD36 (as does *P. falciparum*), but they sequester mainly in the lungs and in adipose tissue rather than in the brain [35]. The fundamental and obvious pathological difference with HCM is that there is marked accumulation of leukocytes and platelets in the venules and capillaries of the brain in the murine model instead of parasitised RBC sequestration (Figure 2). Thus, whereas there is often little or no cytopathological evidence of inflammation in fatal HCM, this is the hallmark of the murine model. In CD36^−/−^ mice infected with *P.berghei* ANKA there is no microvascular sequestration of parasitised erythrocytes but the cerebral syndrome still occurs. Curiously, rather than challenging the relevance of the murine model, this finding was taken to support a dissociation between cerebral sequestration and the causation of human cerebral malaria [35]!

Despite these fundamental differences, the murine and human syndromes are discussed together as if they shared a similar pathology. There is an increasing trend for authors to drop the adjective ‘murine’ from their description of experimental cerebral malaria. A large and remarkably diverse array of interventions has been effective in the mouse model (Table S1 in the supplementary material online). Some make strange bedfellows (anti-TNF antibody and recombinant TNF, oxidants and anti-oxidants, starvation and obesity). Interventions based on an incorrect understanding of pathology can be harmful, as shown previously. Given the considerable differences between the murine and human cerebral syndromes, can we justify evaluating any of these interventions in man, and – if so – which one?

## Responses to interventions

Since the first adjunctive intervention (anti-TNF antibody) in the murine model was evaluated 22 years ago, 48 different potential therapeutic interventions have been evaluated in 45 studies (Table S1 in the supplementary material online). All but four (92%) have either prevented or ameliorated the cerebral syndrome. Of these four exceptions, COX2 inhibition by celecoxib made it worse, whereas indomethacin, simvastatin and anti-apoptotic strategies had no effect. The results of the interventions have usually been dramatic, typically preventing or reducing the cerebral syndrome by more than 50%, and often by 100% [36]. By comparison, in the same period, 11 adjunctive interventions were evaluated in 17 clinical trials in severe human HCM. Only one (with human albumin) was claimed to provide a mortality advantage, and two other interventions (desferrioxamine and pentoxyfilline) claimed acceleration in the recovery from coma. However, for both desferrioxamine and pentoxyfilline, other trials did not confirm the benefit. None of the adjunctive treatments evaluated in HCM before the murine cerebral malaria era was clearly beneficial either. Dexamethasone, pentoxyfilline, anti-TNF antibody and aspirin were evaluated in both mice and men, with dramatic benefits in encephalopathic mice, but not in humans with cerebral malaria. Murine AIDS also protects mice from ‘cerebral malaria’, but HIV infection predisposes to, rather than ameliorates, severe malaria in semi-immune subjects. In summary, whereas nearly all evaluated interventions have been dramatically effective in the murine model, there is no convincing evidence of benefit for any adjunctive intervention in human cerebral malaria, although the jury remains out (awaiting the results of large multicentre trials) on the role of albumin.

## The timing of adjunctive treatment

The relevance of murine ‘cerebral malaria’ intervention results to the treatment of HCM is further challenged by the timing of intervention. In severe falciparum malaria, sequestration of parasitised erythrocytes contributes to the development of coma and subsequent death. There is now good evidence that antimalarial treatment with artemisinin derivatives, that prevent sequestration by killing circulating ring forms of the parasite, significantly reduces mortality [3]. Studies using murine models have typically examined survival benefits following administration of the adjunctive treatment to animals before the development of neurological signs. Once these develop, adjuvant treatments are much less effective. Adjunctive treatments in murine ‘cerebral malaria’ are not evaluated with, or compared to, effective antimalarial drugs, that would be the context of use in treating human falciparum malaria.

## Reconciling clinical and laboratory research on severe malaria

In recent years there has been very little clinicopathological research on HCM, whereas there has been a dramatic increase in laboratory research on the murine ‘model’ (Figure 1). This neuropathological process in CBA or C57BL/6 mice infected with the *P.berghei* ANKA strain has been called ‘cerebral malaria’, a term adopted from clinical medicine because mice with this malaria develop neurological signs. It shares the name, but very little else, with the human disease. The histopathology and the responsivity to interventions are dramatically different. It is clear that the murine disease is primarily an immunopathological process, whereas endothelial activation and dysfunction in HCM are associated with the sequestration of parasitised RBC, and the primary role of immune mechanisms is questionable. Interestingly, pulmonary pathology in the murine model is clearly associated with parasitised RBC sequestration [35,37], and may have a stronger case than the encephalopathy to be a model of human disease. Why some *P. falciparum* parasites sequester preferentially in the human cerebral microvasculature is not known, but this question is unlikely to be answered by an animal model in which white cells, rather than parasitised RBC, accumulate in the brain.

The timing and context of interventions evaluated in the murine model are unrealistic. In the mouse, the interventions are usually assessed before or at the onset of the cerebral syndrome in the absence of antimalarial drugs. In clinical practice, patients are treated immediately with antimalarial drugs. Therefore they seldom present to medical attention ‘before cerebral malaria’ because effective antimalarial treatment with artemisinin derivatives largely prevents it. Most patients who develop cerebral malaria are already unconscious when they present to medical attention, and they are treated immediately with a parenteral antimalarial. No adjunctive treatment is of proven benefit in HCM, whereas nearly everything seems to be dramatically effective in the murine model (including interventions that have been ineffective in HCM). Indeed, it is remarkable just how many completely different interventions prevent the murine neurological syndrome. Of course, it may be that other ineffective interventions were not reported. It must also be conceded that the statistical power of the majority of intervention trials in HCM has been limited. Only four clinical trials recruited more than 100 patients per treatment arm. Therefore, although dramatic benefits have been excluded, small (but clinically important) benefits cannot be excluded by these human clinical studies.

The investigation of murine cerebral malaria has now developed into an independent and self-sustaining episteme. Murine malaria studies usually build on established laboratory research programmes, use genetically homogeneous parasites in genetically homogeneous hosts, provide standardised high quality histopathology on all subjects, give ‘positive’ results, and provide immunological and pathological supporting material, and these appeal to a substantial laboratory-based immunology caucus. The study reports seldom question the relevance of the model to clinical medicine or to the human disease. Despite the marked differences in pathology, and the unrealistic clinical setting evaluated in the murine model, reports of successful interventions in the mouse usually conclude that the intervention may benefit human cerebral malaria. This extrapolation, that may carry considerable weight in the decision to publish, seems unjustified. To illustrate the disconnect, of the eight potential adjunctive treatments for severe malaria now being evaluated in humans (L-arginine, N-acetyl cysteine, albumin, mannitol, erythropoetin, levamisole, activated charcoal, early enteral feeding) only two (erythropoietin and activated charcoal) derive from studies in the murine model (http://www.controlled-trials.com/mrct/search.html).

Animal models can provide very valuable biological information. Our concerns are with this particular model, the use of one term (i.e. ‘cerebral malaria’) to describe two very different clinicopathological processes, and inference from observations in this model to potential interventions in HCM [38,39]. Given the considerable differences between murine ‘cerebral malaria’ and HCM, in pathology and in susceptibility to adjunctive interventions, it is legitimate to question the relevance, and thus the utility of this mouse model.
