# Supplementary material for: The murine cerebral malaria phenomenon
Source: Trends Parasitol. 2010 Jan;26(1):11–5. doi: 10.1016/j.pt.2009.10.007 (PMC2807032; doi:10.1016/j.pt.2009.10.007)
Supplement: Supplementary file 1 [file mmc1.doc]

**Table S1. Adjunctive treatments evaluated in murine ‘cerebral malaria’**

| **Reference** | **Intervention** | **Effect** | **Parasite** | **Mice** | **Author^a^** |
| --- | --- | --- | --- | --- | --- |
| aa | Anti-TNF antibody | Reduced CM  Improved survival | *P. berghei* ANKA | CBA/Ca | Grau *et al.* 1987 |
| ab | Cyclosporine | Reduced CM  Improved survival | *P. berghei* ANKA | CBA/Ca | Grau *et al.* 1988 |
| ac | anti-rGM-CSF and anti-rIL-3 antibodies | Reduced CM | *P. berghei* ANKA | CBA | Grau *et al.* 1988 |
| ad | Antioxidants | Reduced CM | *P. berghei* ANKA | A/J and CBA/H | Thurmwood  *et al.* 1989 |
| ae | Anti Gamma interferon antibody | Reduced CM  Improved survival | *P. berghei* ANKA | CBA/Ca | Grau *et al.* 1989 |
| af | IL1 | Reduced CM  Improved survival | *P. berghei* K 173 | C57Bl/6J | Curfs *et al.* 1990 |
| ag | Anti LFA1 (CD11a) antibody | Reduced CM  Improved survival | *P. berghei* ANKA | CBA/Ca | Grau *et al.* 1991 |
| ah | Pentoxifylline | Reduced CM  Improved survival | *P. berghei* ANKA | CBA/Ca | Kremsner *et al.* 1991 |
| ai | Iloprost | Reduced CM  Improved survival | *P. berghei* ANKA | CBA/Ca | Sliwa *et al.*1991 |
| aj | Fish Oil | Reduced CM  Improved survival | *P. berghei* ANKA | C57B1/6 | Blok *et al.* 1992 |
| aj | Indomethacin | No effect | *P. berghei* ANKA | C57B1/6 | Blok *et al.* 1992 |
| ak | Starvation | Prevention of CM | *P. berghei* ANKA | CBA/T6 | Hunt *et al.* 1993 |
| al | Murine AIDS | Prevention of CM | *P. berghei* ANKA | C57/BL/6 | Eckwalanga  *et al.* 1995 |
| am | Fish oil | Prevention of CM | *P. berghei* ANKA | CBA/CaJ | Levander  *et al.* 1995 |
| an | Dexamethasone | Reduced CM  Improved survival | *P. berghei* ANKA | CBA/T6 and DBA/2J | Neill & Hunt 1995 |
| ao | Fatty acids | Reduced CM | *P. berghei* ANKA | C57/BL/6 | Moumaris  *et al.* 1995 |
| ap | Coinfection with *B.pahangi* | Reduced CM  Improved survival | *P. berghei* | CBA/J | Yan  *et al.* 1997 |
| aq | CD4 or CD8 depletion | Reduced CM | *P. berghei* K 173 | C57B1/6J and C57B1/10 | Hermsen  *et al.* 1997 |
| ar | Recombinant IL-10 | Reduced CM | *P. berghei* ANKA | CBA/J | Kossodo  *et al.* 1997 |
| as | Splenectomy | Reduced CM  Improved survival | *P. berghei* K 173 | C57BL/6J | Hermsen  *et al.* 1998 |
| at | Thiolated recombinant TNF (rhTNF alpha-AT) | Reduced CM  Improved survival | *P. berghei* K173 | C57BL/6J | Postma *et al.* 1999 |
| au | Liposomal recombinant TNF | Reduced CM  Improved survival | *P. berghei* K173 | C57BL/6J | Postma *et al.* 1999 |

|  | **Intervention** | **Effect** | **Parasite** | **Mice** | **Author** |
| --- | --- | --- | --- | --- | --- |
| av | Dichloroacetate | Improved survival | *P. berghei* ANKA | CBA/T6 | Rae *et al.* 2000 |
| aw | Immunisation with synthetic GPI | Prevents CM and death | *P.berghei* ANKA | C57BL6 | Schofield  *et al.* 2002 |
| ax | Anti CD 41 | Reduced CM  Improved survival | *P. berghei* ANKA | C57BL/6 | Sun  *et al.* 2003 |
| ax | Anti CD 61 | Reduced CM  Improved survival | *P. berghei* ANKA | C57BL/6 | Sun  *et al.* 2003 |
| ay | Celecoxib | Earlier onset of CM | *P. berghei* ANKA | CBA or C57BL6 | Ball *et al.* 2004 |
| az | kynurenine-3-hydroxylase inhibitor | Reduced CM  Improved survival | *P. berghei* ANKA | C57BL/6J | Clark  *et al.* 2005 |
| ba | Thalidomide | Improved survival | *P. berghei* ANKA | CBA | Muniz-Junqueira  *et al.* 2005 |
| bb | Erythropoietin | Improved survival | *P. berghei* ANKA | CBA/J | Kaiser *et al.* 2006 |
| bc | Nitric oxide | Reduced CM  Improved survival | *P. berghei* ANKA | C57BL/6 | Gramaglia *et al.* 2006 |
| bd | anti-BTLA mAb | Reduced CM | *P .berghei* ANKA | C57BL/6 | Lepenies *et al.* 2007 |
| be | Carbon monoxide | Reduced CM  Improved survival | *P. berghei* ANKA | C57BL/6 | Pamplona *et al.* 2007 |
| bf | Recombinant human IFN-alpha | Reduced CM  Improved survival | *P. berghei* ANKA | C57BL/6 | Vigario *et al.* 2007 |
| bg | anti CD25 antibody | Reduced CM  Improved survival | *P. berghei* ANKA | CBA and C57BL/6 | Amante *et al.* 2007 |
| bh | CD8 T cell depletion | Prevention or attenuation of CM | *P. berghei* ANKA | CBA and C57BL/6 | Randall *et al.* 2008 |
| bi | Guanylhydrazone CNI-1493 | Reduced CM  Improved survival | *P. berghei* ANKA | C57BL/6 | Specht *et al.* 2008 |
| bj | Antibody blockade of C5a or C5a receptor (C5aR) | Reduced CM  Improved survival | *P. berghei* ANKA | C57BL/6 | Patel *et al.* 2008 |
| bk | Pantethene | Reduced CM  Improved survival | *P. berghei* ANKA | CBA/J | Penet *et al.* 2008 |
| bl | Simvastatin | No effect | *P. berghei* ANKA | C57BL/6 | Kobbe *et al.* 2008 |
| bm | Aspirin | Reduced CM  Improved survival | *P. berghei* ANKA | C57BL/6 | Srivastava *et al.* 2008 |
| bm | Clopidrogrel | Reduced CM  Improved survival | *P. berghei* ANKA | C57BL/6 | Srivastava *et al.* 2008 |
| bn | Anti-apoptotic strategies | No effect | *P. berghei* ANKA | C57BL/6 | Helmers *et al.* 2008 |
| bo | Obesity | Prevented CM  Improved survival | *P. berghei* ANKA | C57BL/6 | Robert *et al.* 2008 |
| bp | Hyperbaric oxygen | Prevented CM  Improved survival | *P. berghei* ANKA | C57BL/6 | Blanco *et al.* 2008 |
| bq | Glatiramer | Reduced CM  Improved survival | *P. berghei* ANKA | C57BL/6 | Lackmer *et al.* 2009 |
| br | IP 10 antibodies | Reduced CM  Improved survival | *P. berghei* ANKA | C57BL/6 | Nie *et al.* 2009 |
| bs | Rosiglitazone | Reduced CM  Improved survival | *P. berghei* ANKA | C57BL/6 | Serghides *et al.* 2009 |

^a^Full references for adjunctive interventions are listed below

**References to cerebral malaria interventions or adjunctive treatments**

**(from Table S1; reference in brackets)**

1. **Studies in the murine “model”**
2. (aa) Grau, G.E. *et al*. (1987) Tumor necrosis factor (cachectin) as an essential mediator in murine cerebral malaria. *Science.* 237, 1210-1212
3. (ab) Grau, G.E. *et al*. (1987) Prevention of murine cerebral malaria by low-dose cyclosporin A. *Immunology*  61, 521-525
4. (ac) Grau, G.E. *et al*. (1988) Prevention of experimental cerebral malaria by anticytokine antibodies. Interleukin 3 and granulocyte macrophage colony-stimulating factor are intermediates in increased tumor necrosis factor production and macrophage accumulation. *J. Exp. Med*. 168, 1499-1504
5. (ad) Thumwood, C.M. *et al*. (1989) Antioxidants can prevent cerebral malaria in *Plasmodium berghei*-infected mice. *Br. J. Exp. Pathol.* 70, 293-303
6. (ae) Grau G.E. *et al*. (1989) Monoclonal antibody against interferon gamma can prevent experimental cerebral malaria and its associated overproduction of tumor necrosis factor. *Proc. Natl. Acad. Sci. U. S. A.*  86, 5572-5574
7. (af) Curfs, J.H. *et al*. (1990) Low dosages of interleukin 1 protect mice against lethal cerebral malaria. *J. Exp. Med.* 172, 1287-1291
8. (ag) Grau, G.E. *et al*. (1987) Late administration of monoclonal antibody to leukocyte function-antigen 1 abrogates incipient murine cerebral malaria. *Eur. J. Immunol.* 21, 2265-2267
9. (ah) Kremsner, P.G. *et al*. (1991) Pentoxifylline prevents murine cerebral malaria. *J. Infect. Dis*. 164, 605-608
10. (ai) Sliwa, K. *et al*. (1991) Prevention of murine cerebral malaria by a stable prostacyclin analog. *Infect. Immun.* 59, 3846-3848
11. (aj) Blok, W.L. *et al*. (1992) Dietary fish-oil supplementation in experimental gram-negative infection and in cerebral malaria in mice. *J. Infect. Dis*. 165, 898-903
12. (ak) Hunt, N.H. *et al*. (1993) Amelioration of murine cerebral malaria by dietary restriction. *Parasitology* 107, 471-476
13. (al) Eckwalanga, M. *et al*. (1994) Murine AIDS protects mice against experimental cerebral malaria: down-regulation by interleukin 10 of a T-helper type 1 CD4+ cell-mediated pathology. *Proc. Natl. Acad. Sci. U. S. A.* 91, 8097-8101
14. (am) Levander, O.A. *et al*. (1995) Protection against murine cerebral malaria by dietary-induced oxidative stress. *J. Parasitol.* 81, 99-103
15. (an) Neill, A.L. and Hunt, N.H. (1995) Effects of endotoxin and dexamethasone on cerebral malaria in mice. *Parasitology* 111, 443-454
16. (ao) Moumaris, M. *et al*. (1995) Effect of fatty acid treatment in cerebral malaria-susceptible and nonsusceptible strains of mice. *J. Parasitol*. 81, 997-999
17. (ap) Yan, Y. *et al*. (1997) Down-regulation of murine susceptibility to cerebral malaria by inoculation with third-stage larvae of the filarial nematode *Brugia pahangi*. *Parasitology* 114 , 333-338
18. (aq) Hermsen, C. *et al*. (1997) Depletion of CD4+ or CD8+ T-cells prevents Plasmodium berghei induced cerebral malaria in end-stage disease. *Parasitology* 114 , 7-12
19. (ar) Kossodo, S. *et al*. (1997) Interleukin-10 modulates susceptibility in experimental cerebral malaria. *Immunology*. 91, 536-544
20. (as) Hermsen, C.C. *et al*. (1998) Convulsions due to increased permeability of the blood-brain barrier in experimental cerebral malaria can be prevented by splenectomy or anti-T cell treatment. *J. Infect. Dis.* 178, 1225-1227
21. (at) Postma, N.S. *et al*. (1999) Thiolated recombinant human tumor necrosis factor-alpha protects against *Plasmodium berghei* K173-induced experimental cerebral malaria in mice. *Antimicrob. Agents. Chemother.* 43, 1027-1033
22. (au) Postma, N.S. *et al*. (1999) Treatment with recombinant human tumor necrosis factor-alpha reduces parasitemia and prevents *Plasmodium berghei* K 173 - Induced experimental cerebral malaria in mice. *Parasitology* 118, 7-15
23. (av) Rae, C. *et al*. (2000) Dichloroacetate (DCA) reduces brain lactate but increases brain glutamine in experimental cerebral malaria: a 1H-NMR study. *Redox Rep.* 5, 141-143
24. (aw) Schofield, L. (2002) Synthetic GPI as a candidate anti-toxic vaccine in a model of malaria. *Nature* 418, 785-789
25. (ax) Sun, G. *et al*. (2003) Inhibition of platelet adherence to brain microvasculature protects against severe *Plasmodium berghei* malaria. *Infect. Immun* 71, 6553-61.
26. (ay) Ball, H.J. *et al*. (2004) Cyclooxygenase-2 in the pathogenesis of murine cerebral malaria. *J. Infect. Dis.* 189, 751-758
27. (az) Clark, C.J. *et al*. (2005) Prolonged survival of a murine model of cerebral malaria by kynurenine pathway inhibition. *Infect. Immun.* 73, 5249-5251
28. (ba) Muniz-Junqueira, M.I. *et al*. (2005) Thalidomide influences the function of macrophages and increases the survival of *Plasmodium berghei*-infected CBA mice. *Acta .Trop.* 94, 128-138
29. (bb) Kaiser, K. *et al*. (2006) Recombinant human erythropoietin prevents the death of mice during cerebral malaria. *J. Infect. Dis*. 193, 987-995
30. (bc) Gramaglia, I. *et al*. (2006) Low nitric oxide bioavailability contributes to the genesis of experimental cerebral malaria. *Nat. Med.* 12, 1417-1422
31. (bd) Lepenies, B. *et al*. (2007) Ligation of B and T lymphocyte attenuator prevents the genesis of experimental cerebral malaria. *J. Immunol.* 179, 4093-4100
32. (be) Pamplona, A. *et al*. (2007) Heme oxygenase-1 and carbon monoxide suppress the pathogenesis of experimental cerebral malaria. *Nat. Med.* 13, 703-710
33. (bf) Vigário, A.M. *et al*. (2007) Recombinant human IFN-alpha inhibits cerebral malaria and reduces parasite burden in mice. *J. Immunol.* 178, 6416-6425
34. (bg) Amante, F.H. *et al*. (2007) A role for natural regulatory T cells in the pathogenesis of experimental cerebral malaria. *Am. J. Pathol.* 171, 548-559
35. (bh) Randall, L.M. *et al*. (2008) Common strategies to prevent and modulate experimental cerebral malaria in mouse strains with different susceptibilities. *Infect. Immun.* 76, 3312-3320
36. (bi) Specht, S. *et al*. (2008) The guanylhydrazone CNI-1493: an inhibitor with dual activity against malaria-inhibition of host cell pro-inflammatory cytokine release and parasitic deoxyhypusine synthase. *Parasitol. Res.* 102, 1177-1184
37. (bj) Patel, S.N. *et al*. (2008) C5 deficiency and C5a or C5aR blockade protects against cerebral malaria. *J. Exp. Med.* 205, 1133-1143
38. (bk) Penet, M.F. *et al*. (2008) Protection against cerebral malaria by the low-molecular-weight thiol pantethine. *Proc. Natl. Acad. Sci. U. S. A.* 105, 1321-1326
39. (bl) Kobbe, R. *et al*. (2008) Simvastatin treatment shows no effect on the incidence of cerebral malaria or parasitemia during experimental malaria. *Antimicrob. Agents. Chemother.* 52, 1583-1584
40. (bm) Srivastava, K. *et al*. (2008) Platelet factor 4 mediates inflammation in experimental cerebral malaria. *Cell. Host Microbe.* 4, 179-187
41. (bn) Helmers, A.J. *et al*. (2008) Failure of two distinct anti-apoptotic approaches to reduce mortality in experimental cerebral malaria. *Am. J. Trop. Med. Hyg.* 79, 823-825
42. (bo) Robert V. *et al*. (2008) Malaria and obesity: obese mice are resistant to cerebral malaria. *Malar. J.* 7, 81
43. (bp) Blanco, Y.C. *et al*. (2008) Hyperbaric oxygen prevents early death caused by experimental cerebral malaria. *PLoS One.* 3, e3126
44. (bq) Lackner, P. *et al*. (2009) Glatiramer acetate reduces the risk for experimental cerebral malaria: a pilot study, Part A. *Malar. J.* 8, 36
45. (br) Nie, C.Q. *et al*. (2009) IP-10-mediated T cell homing promotes cerebral inflammation over splenic immunity to malaria infection. *PLoS Pathog.* 5, e1000369
46. (bs) Serghides, L. *et al*. (2009) Rosiglitazone modulates the innate immune response to *Plasmodium falciparum* infection and improves outcome in experimental cerebral malaria. *J. Infect. Dis.* 199, 1536-1545
47. **Studies and reviews of studies in humans since 1980**
48. (ca) Warrell, D.A. *et al*. (1982) Dexamethasone proves deleterious in cerebral malaria. A double blind trial in 100 comatose patients.  *N. Engl. J. Med.* 306, 313-319
49. (cb) Hoffman, S. L. *et al*. (1988) High-dose dexamethasone in quinine-treated patients with cerebral malaria: a double-blind, placebo-controlled trial.  *J. Infect. Dis*. 158, 325-331
50. (cc) Hemmer, C.J. *et al*. (1991) Neither heparin nor acetylsalicylic acid influence the clinical course in human *Plasmodium falciparum* malaria:a prospective randomized study. *Am. J Trop. Med. Hyg.* 45*,*  608-612
51. (cd) Taylor, T.E. *et al*. (1992) Intravenous immunoglobulin in the treatment of paediatric cerebral malaria. *Clin. Exp. Immunol*. 90, 357-362
52. (ce) Gordeuk, V. *et al*. (1992) Effect of iron chelation therapy on recovery from deep coma in children with cerebral malaria.  *N. Engl. J. Med.*  327, 1473-1477
53. (cf) Di Perri, G. *et al*. (1995) Pentoxifylline as a supportive agent in the treatment of cerebral malaria in children. *J. Infect. Dis*. 171, 1317-1322
54. (cg) van Hensbroek, M.B. *et al*. (1996) The effect of a monoclonal antibody to tumor necrosis factor on survival from childhood cerebral malaria. *J. Infect. Dis*. 174, 1091-1097
55. (ch) Hemmer, C.J. *et al*. (1997) Supportive pentoxifylline in falciparum malaria: no effect on tumour necrosis factor alpha levels or clinical outcome: a prospective, randomized, placebo controlled study. *Am. J. Trop. Med. Hyg.* 56, 397-403
56. (ci) Looareesuwan, S. *et al*. (1998) Pentoxifylline as an ancillary treatment for severe falciparum malaria in Thailand. *Am. J. Trop. Med. Hyg.* 58, 348-353
57. (cj) Thuma, P.E. *et al*. (1998) Effect of iron chelation therapy on mortality in Zambian children with cerebral malaria. *Trans. R. Soc. Trop. Med. Hyg.* 92, 214-218
58. (ck) Looareesuwan, S. *et al*. (1999) Polyclonal anti-tumor necrosis factor alpha Fab used as an ancilliary treatment for severe malaria. *Am. J. Trop. Med. Hyg.* 61, 26-33
59. (cl) Smith, H.J. and Meremikwu, M. (2000) Iron chelating agents for treating malaria. *Cochrane. Database Syst. Rev.* 2*,* CD001474
60. (cm) Mohanty, D. *et al*. (2002) Deferiprone L1 as an adjuvant therapy for *Plasmodium falciparum* malaria. *Indian J. Med. Res.*  115*,* 17-21
61. (cn) Watt, G. *et al*. (2002) A pilot study of N-acetylcysteine as adjunctive therapy for severe malaria. *Q. J. Med.*  *95* , 285-290
62. (co) Das, B.K. *et al*. (2003) Pentoxifylline adjuvant improves prognosis of human cerebral malaria in adults. *Trop. Med. Int. Health* 8, 680-684
63. (cp) Okoromah, C.A. and Afolabi, B.B. (2004) Mannitol and other osmotic diuretics as adjuncts for treating cerebral malaria. *CochraneDatabase Syst. Rev.* 4, CD004615
64. (cq) Enwere, G. (2005) A review of the quality of randomized clinical trials of adjunctive therapy for the treatment of cerebral malaria. *Trop. Med. Int. Health* 10, 1171-1175
65. (cr) Maitland, K. *et al*. (2005) Randomized trial of volume expansion with albumin or saline in children with severe malaria: preliminary evidence of albumin benefit. *Clin. Infect. Dis.* 40, 538-545
66. (cs) Mohanty, S. *et al*. (2006) Adjuvant therapy in cerebral malaria. *Indian. J. Med. Res.* 124, 245-260
67. (ct) Akech, S. *et al*. (2006) Volume expansion with albumin compared to gelofusine in children with severe malaria: results of a controlled trial. *PLoS. Clin. Trials* 1, e21
68. (cu) Namutangula, B. *et al*. (2007) Mannitol as adjunct therapy for childhood cerebral malaria in Uganda: a randomized clinical trial. *Malar. J.*  6, 138
69. (cv) Charunwatthana, P. *et al*. (2009) N-acetylcysteine as adjunctive treatment in severe malaria: a randomized, double-blinded placebo-controlled clinical trial. *Crit. Care. Med.* 37, 516-522
